# Effectiveness of a bio-catalytic agent used in the bioremediation of crude oil-polluted seawater

**DOI:** 10.1016/j.heliyon.2021.e06926

**Published:** 2021-04-30

**Authors:** Glenda Teran-Cuadrado, Efrain Polo-Cuadrado

**Affiliations:** a Chemical Engineering Department, University of Atlantico, Puerto Colombia, Atlántico, Colombia; b Organic Syntesis Laboratory, Natural Resources Chemistry Institute, University of Talca, Talca, Maule, 3460000, Chile

**Keywords:** Non-ionic surfactant, Bioremediation, Crude oil, Seawater, Bio-catalyst, Environmental impacts

## Abstract

Oil spillage contamination has been one of the most common and challenging problems in marine ecosystems over the years due to frequent petroleum exploitation, washing, and transportation activities. The use of nature-derived surfactants has become an attractive approach to restore the sites affected by oil spillage. Several studies have demonstrated that nutrient addition is an efficient strategy to enhance oil biodegradation since microorganisms can use petroleum hydrocarbons as their carbon and energy source, thus favoring and increasing the hydrocarbons degradation rate. This study aimed to assess the effectiveness of a commercial bio-catalytic agent used in the biological remediation of crude oil-contaminated sites through the qualitative analysis of its properties. The tests applied to this bio-catalyst showed excellent results. For instance, the emulsification (E_24_) and critical micellar concentration (CMC) assays displayed average values of 74.47% and 40 mg L^−1^, respectively. A significant reduction of Chemical Oxygen Demand (COD), turbidity, and Total Petroleum Hydrocarbon Content (TPHC) were observed in all the samples with bio-catalytic agent solution and aeration system. The best water quality was achieved by the sample with the highest concentration (10000 ppm) of bio-catalytic agent solution. It displayed a Total Petroleum Hydrocarbon removal efficiency (RTPH) of 81.537% after 30 days of the remediation time.

## 1 Introduction

The hydrocarbon industry has reached a considerable growth during the last decades, and nowadays, it is one of the most essential links in the economic and social development worldwide. The increase in total demand for energy and water has led to the implementation of water-intensive forms of power generation and energy-intensive platforms of water production, primarily driven by population growth. Consequently, this excessive energy consumption has triggered the frequent exploitation of hydrocarbon reserves without considering the environmental impacts on terrestrial, aquatic and aerial ecosystems (Leahy and Colwell, 1990). The limited biodegradation capacity of petroleum-derived hydrocarbons and their low reactivity represent a significant threat to the environment, owing to the high level of toxicity and inhibition to plant and animal growth and their mutagenic and carcinogenic characteristics (United Nations Educational Scientific and Cultural Organization, 2019). The above statement agrees with what Adams et al. (2008) affirm: "Oil contamination in bodies of water causes an impermeable film that quickly affects gas exchange and the passage of sunlight, giving way to the rupture of the food chain and a series of simultaneous physical and chemical changes, which make the natural degradation process slower, inefficient, and toxic”. This can produce substantial structural changes in the phytoplankton communities and the rest of marine fauna and flora (Asimea and Sam-Wobo, 2011).

On average, roughly nine million tons of petroleum hydrocarbons are discharged into aquatic ecosystems all over the world each year, especially into marine waters and estuaries (Torres, 2003). Indeed, the oil spillage in the Gulf of Mexico, considered one of the most catastrophic events in history, released about 600000 tons of crude oil into the sea (Dell’Anno et al., 2018). The largest source of pollution by hydrocarbons and their derivatives in marine environments comes from routine ship/boat washing activities, natural oil leaks on the sea surface, and accidents during the exploration and transportation of crude oil (Marques-Junior et al., 2009).

Although conventional oil removal methods such as physical extraction are often the first response option, they unlikely achieve a complete cleanup of oil spills. These techniques often use traditional physical methods such as grease traps, evaporation, and separation with ultrafiltration membrane. Additionally, chemical methods like gas and ozone injection, chemical precipitation, ion exchange are usually applied to treat this type of contamination. The problem is that these methods require high investment for their implementation and operation, and in some cases, end up transferring pollutants to other media.

The negative impacts on the environment, food safety, human health, the integrity of fauna and flora species, and the stability of petroleum hydrocarbons make it necessary to develop alternative treatment methods to the physical and chemical methodologies. These must be more effective, environmentally-friendly, and faster compared to natural biodegradation processes.

Bioremediation processes are an alternative technology that meets these requirements and have been on the rise since the early 1990s when they were popularized as the ultimate solution to oil spills (Hoff, 2003). This technology seeks to recover contaminated sites using organisms (plants, fungi, bacteria, or enzymes). For this purpose, it considers the metabolic processes of the microorganisms and how they will transform the pollutant into biomass and carbon dioxide (mineralization) (Gamba and Pedraza, 2018). This biological remediation uses bio-stimulation and bio-augmentation as potential strategies to hasten natural attenuation or biodegradation (Ladousse and Tramier, 1991). The last advances in sustainable technologies have led to the use of surfactants, which are chemical compounds with high surface activity (Gervajio et al., 2020). They can improve the conditions and results of bioremediation. Bio-surfactants are a kind of surfactant naturally produced by microorganisms or extracted from plants or animals. Owing to their biodegradability and low toxicity, they are preferred to remediate petroleum hydrocarbon-contaminated sites (Silva et al., 2014).

The possibility of synthesizing this type of compound from low-cost sources and industrial waste has made them ideal for treating areas affected by oil. Furthermore, their outstanding biodegradation capability, detoxification of industrial effluents, and high effectiveness under conditions of extreme temperature, pH, and salinity manifest their versatility (Pirôllo, 2006). Moreover, the addition of surfactants is paramount during the preparation of hybrid nanofluids since they can improve the thermophysical and rheological properties of this type of nanofluids. The application of them enables the longer stability period of hybrid nanofluids with an uniform dispersion of nanoparticles, increasing the thermal conductivity and decreasing the viscosity (Shah and Koten, 2020). These characteristics have driven to a significant production of natural surfactants, as supported by data from Campos et al. (2013), who point out that in 2012 these compounds represent 3.5 million tons of the total of surfactants produced worldwide, which is translated into the generation of 6588 million dollars per year.

The importance of this project, developed on a laboratory scale, lies in analyzing the bio-catalytic agent quality through the monitoring of degradation behavior of hydrocarbons present in seawater as a function of the Total Petroleum Hydrocarbon Content (TPHC) and some physical and chemical parameters such as Chemical Oxygen Demand, Dissolved Oxygen, turbidity, among others.

## 2 Materials and methods

### 2.1 Chemicals

All reactants used were technical, analytical, pure, or reagent grades without being modified in their original composition. Hydrochloric acid (HCl, 37 wt.%), glycerol (C_3_H_8_O_3,_ 99 wt.%), sodium dodecyl sulfate (SDS) (NaC_12_H_25_SO_4,_ 90 wt.%), ethyl alcohol (C_2_H_5_OH, 99 wt.%), and methanol (CH_3_OH, 99 wt.%) were provided by Labsynth. β-mercaptoethanol (C_2_H_6_OS, 99 wt. %), tris (hydroxymethyl) aminomethane-HCl (CNH_2_(CH_2_OH)_3,_ 99 wt.%), bromophenol blue (C_19_H_10_Br_4_O_5_S, 99 wt.%), acrylamide (C_3_H_5_ON, 99 wt.%), tris(2-carboxyethyl) phosphine (C_9_H_15_O_6_P, 99 wt.%), formic acid (CH_2_O_2,_ 95 wt.%), sodium form (NaCOOH, 99 wt.%), potassium acid phthalate (C_8_H_5_KO_4_), and silver nitrate (AgNO_3,_ 98 wt.%) were sold by Sigma-Aldrich. Ethylenediaminetetraacetic acid (EDTA) (C_10_H_16_N_2_O_8,_ 99 wt. %) and kerosene (C_12_H_26,_ 99 wt. %) were provided by Neon and Natrielli, respectively. Potassium dichromate (K_2_Cr_2_O_7,_ 99.5 wt. %), mercury sulfate (Hg_2_SO_4,_ 98 wt. %), silver sulfate (Ag_2_SO_4,_ 99 wt.%), and potassium chromate (K_2_CrO4, 99.5 wt.%) were purchased from PanReac. Sulfuric acid (H_2_SO_4,_ 95–98 wt. %) and hexane (C_6_H_14,_ ≥ 98.5 wt. %) were obtained from J.T.Baker.

### 2.2 Sample preparation and bioremediation study

The oil-polluted water used in this study was obtained by spilling a specific volume of Vasconia heavy crude oil on natural seawater. The crude oil sample was collected from an oil & gas refining company in Colombia, and the seawater was obtained directly from the sea in a coastal town in Colombia. The properties of the crude oil sample were: API gravity (24.27 API°), specific gravity at 15 °C (0.908), viscosity at 40 °C (22 cSt), Sulphur content (0.833 wt.%), Reid Vapor Pressure (RVP) (21.99 kPa), and Flash Point (0 °C).

The bio-catalyst was supplied by its official distributor in Colombia. The product composition comprises sodium benzoate, imidazolidinyl urea, diazolidinyl urea, a fermentation supernatant derived from a Saccharomyces cerevisiae culture, and a non-ionic surfactant that was extracted from plants and minerals. It can belong to, but is not limited to, polyether non-ionic surfactants comprising fatty alcohols, alkyl phenols, fatty acids, and fatty amines which have been ethoxylated; polyhydroxyl non-ionic (polyols) typically comprising sucrose esters, sorbital esters, alkyl glucosides, and polyglycerol esters which may or may not be ethoxylated (Dale and Hill, 1998). According to the supplier, this product is a totally safe and completely soluble in water bio-catalytic degrader of organic waste materials. This bio-catalyst is biodegradable regarding the positive results of the Organization for the Economic Co-operation and Development (OCDE) 302B test for ready biodegradability. Also, eco-toxicity characteristics were tested for microorganisms and aquatic organisms on an acute basis ((LC_50_/EC_50_ between 1 and 10 mg/L in the most sensitive species tested). These results validate the non-toxicity nature of this product(Neozyme International, 2015).

The crude oil-polluted seawater samples were prepared into six beakers by adding 0.5 mL of Vasconia crude oil to 700 mL of seawater in each glass vessel. The seawater bottles were stored in six translucid glass vessels and then left to stand for four (4) days to allow the indigenous micro-organisms to acclimatize to their new environment. Table 1 shows the various samples and their constituents.

**Table 1:** Samples used and their components.

Sample	Components
A (Control)	Crude oil and seawater only (640 mg L^−1^)
B	Seawater, bio-catalytic agent solution (2167.39 mg L^−1^), and crude oil (640 mg L^−1^)
C	Seawater, bio-catalytic agent solution (10000 mg L^−1^) and crude oil (640 mg L^−1^).
D (Control)	Seawater, bio-catalytic agent solution (2167.39 mg L^−1^) and aeration system.
E	Seawater, bio-catalytic agent solution (2167.39 mg L^−1^), crude oil (640 mg L^−1^) and aeration system.
F	Seawater, bio-catalytic agent solution (10000 mg L^−1^), crude oil (640 mg L^−1^) and aeration system.

Samples labeled B, D, and E were amended by the addition of 1.3 mL of bio-catalyst solution concentrated at 2167.39 mg L^−1^ (bio-catalyst) to each mixture of the samples, following the instructions stated on the product data-sheet. On the other hand, the same volume of bio-catalytic agent solution but with a concentration of 10000 mg L^−1^ was added to samples labeled C and F to evaluate the effect of the dosage of the bio-catalytic agent on the effectiveness of the bioremediation process. This concentration was selected regarding the information provided by the manufacturing company of the product. They state that this bio-catalyst can be applied to TPH contaminated soil, shorelines, and beaches at dilutions of 0.2%–2% v/v.

All the experimental set-up vessels were stored at 25 °C and average relative humidity of 64.5%. During the incubation time, the temperature and relative humidity percentages were continuously controlled by a sensor (PCE Instruments, PCE-P18L, and model). The samples labeled D, E and F were agitated uninterruptedly for aeration and mixing to increase contact between the indigenous microbial consortium, nutrients, and contaminated water.

The other samples labeled A, B, and C were subjected to an agitation system in a magnetic stirrer. Samples from each vessel were analyzed on days 0,4,9,16,23, and 30. The following bioremediation indicating parameters in the polluted water were monitored in the study of remediation; Chemical Oxygen Demand (COD), Turbidity, Total Petroleum Hydrocarbon Content (TPHC), pH, and Dissolved Oxygen (DO).

Considering that the growth of aerobic mesophylls microorganisms is propitious in polluted and aerated medium, the Total Microbial Count (TMC) was measured only on the crude oil-contaminated samples with an aeration system (E and F). Likewise, it is worth pointing out that due to the absence of external bacteria consortium (bio-augmentation), the measurements of TMC were only taken at the beginning and the end of the experiment to observe a significant difference in the bacterial growth.

### 2.3 Methods used in analytical studies

The following methods were quite relevant to determine the bio-catalyst quality and predict its more possible degradation behavior. The qualitative characterization was the criteria to decide to analyze the physicochemical properties of the seawater and confirm the expected performance of the product.

#### 2.3.1 Drop-collapse test

This test was executed according to the experimental method described by Jain et al. (1991), and adapted by Bodour and Miller-Maier (1998). A clean flat surface was used to carry out the experiment, and the holes in there were filled with 5 μL of vegetable oil and 5 μL of bio-catalyst solution were added to the oil surface. After that, the behavior of the drop was inspected for 1 min. If the drop retains its shape, it indicates a negative result, while if the drop collapses mean a positive response.

#### 2.3.2 Oil-spreading assay

5 mL of distilled water were poured into a 15 cm diameter Petri dish, followed by the addition of 100 μL of Bazu oil, supplied by a Brazilian refinery company, to the surface of the water to form a thin layer of oil. About 10 μL of the bio-catalytic agent solution was carefully added to the center of the formed oil layer, and the diameter of the cleaned area was measured (Moro et al., 2018). If the action of bio-catalytic agent is significant, the oil layer will be displaced, resulting in a decontaminated zone free of crude oil. The diameter measurement is closely related to the surfactant activity (Pornsunthorntawee et al., 2008).

#### 2.3.3 Emulsification assay (E_24_)

The emulsifying activity of the studied product was measured using the method described by Cooper and Goldenberg (1987). The test was realized by mixing 2 mL of kerosene with an equal volume of a bio-catalytic agent, which was previously stirred in a vortex type agitator for 2 min and left to stand for twenty-four (24) hours. The emulsification index was calculated as the ratio between the height of the emulsion of foam layer (cm) and the total height of the liquid in the tube (cm), multiplied by 100.

#### 2.3.4 Critical Micelle Concentration (CMC)

This test required the preparation of different dilutions of a bio-catalytic agent in distilled water. The changes in surface tension were measured in a Gibertini brand digital tensiometer at a temperature of 25 °C (Moro et al., 2018). The CMC value of the bio-catalytic agent was determined graphically by the surface tension inflection point (Y-axis) versus the bio-catalytic agent concentration (X-axis) (Nitschke and Pastore, 2006).

The tensiometer measurements were taken by immersion of a coverslip below the surface of the surfactant solution (1 mm approximately), which was slowly extracted, then the maximum force was measured and registered. The distilled water of 96% purity was used as standard.

#### 2.3.5 Determination of pH

The pH values were obtained using a pH and temperature probe (HACH® HQ40D, model) coupled with a multiparameter of continuous reading that worked under the potentiometric method described by the 4500-H APHA Standard Method (American Health Public Association, 2017a). The uncertainty associated with the equipment reading was ±0.016(Standard Deviation).

#### 2.3.6 Determination of turbidity

The Standard Methods 2130 B protocol (American Health Public Association, 1992) was followed to analyze the turbidity during the remediation time. The turbidity of the samples was determined using a turbidimeter (TN400/TUR-001) suitable for readings between 0.02 - 800 NTU. The uncertainty associated with the equipment reading was ±0.053(Standard Deviation).

#### 2.3.7 Determination of dissolved oxygen

The Dissolved Oxygen was measured using a luminescence Oxygen probe (HACH® (LDO) LDBO101, model), coupled with a multiparameter with continuous reading. The dissolved Oxygen content values were in the range of 0.01 up to 20 mgO_2_ L^−1^. The uncertainty associated with the equipment reading was ±0.048(Standard Deviation).

#### 2.3.8 Determination of Chemical Oxygen Demand

COD concentration was determined by the spectrophotometric method 5220D (closed reflux spectrophotometric method), described in the Standard Methods for Examination of Water and Wastewater (American Health Public Association, 2017b).

According to the procedure, the digestion tubes were prepared by adding 1.5 mL of digestion solution and 3.5 mL of catalyst solution. They were left in agitation for two (2) days until complete dissolution. Thenceforth, 2.5 mL of sample was added to the test tube and hermetically sealed, with subsequent agitation for the homogenization of all components inside the digestion tube. The tubes were taken to a thermoreactor (HANNAH Instruments® 839800, model) for two (2) hours at 150 °C. After the digestion process, the tubes were removed from the thermoreactor and get cold up to room temperature. Hereafter, the COD concentrations were taken in Oxygen mg L^−1^ using a spectrophotometer (GENESYS™ 10S UV VIS, model). The uncertainty associated with the equipment reading was ±0.125(Standard Deviation).

#### 2.3.9 Determination of Total Petroleum Hydrocarbon Content (TPHC)

The TPHC values were measured and monitored according to the gravimetric method 1664A of the USEPA (The United States Environmental Protection Agency), which involves a liquid-liquid extraction with hexane followed by the concentration of Total Petroleum Hydrocarbon (TPH) in a roto-evaporator system. The results of this analysis were obtained as the difference between the final weight of the flask and the initial weight of the dry and empty flask in mg, divided by the initial volume of the sample in Litres (United States Environmental Protection Agency, 2000). The uncertainty associated with the equipment reading was ±0.015(Standard Deviation).

The Chemical Oxygen Demand and the TPHC were defined as the following parameters: COD percentage removal efficiency (%CODR) and Total Petroleum Hydrocarbon Percentage Removal (%TPHR), respectively (See Eqs. (1) and (2)).

(1) $$\text{\%RCOD}=\left(\frac{{\text{COD}}_{\text{i}}-{\text{COD}}_{\text{f}}}{{\text{COD}}_{\text{i}}}\right)\text{∗}100$$

where %RCOD is the percentage of Chemical Oxygen Demand Removal Efficiency; COD_f,_ is the COD concentration (ppm) at the end of the experiment, and COD_i,_ is the COD concentration (ppm) at the beginning of the experiment

(2) $$\text{\%RTPH}=\left(\frac{{\text{TPH}}_{\text{i}}-{\text{TPH}}_{\text{f}}}{{\text{TPH}}_{\text{i}}}\right)\text{∗}100$$

where %RTPH is the percentage of TPH clean-up or removal efficiency; TPH_f_ is the TPH concentration (ppm) at the end of the experiment, and TPH_i_ is the TPH concentration (ppm) at the beginning of the experiment.

#### 2.3.10 Determination of TMC

The count of aerobic mesophilic microorganisms was performed according to the NTC 4519 method (Instituto Colombiano de Normas Técnicas y Certificación (ICONTEC), 2013). To quantify the viable microorganisms, the sample was inoculated in a culture medium and poured into a Petri dish. An automatic spiral plater (NF V08-100) was used to incubate the sample at 35 °C for seventy-two (72) hours. The TMC values were collected from the number of colonies counted in the Petri dish per 100 mL of sample.

## 3 Results and discussions

### 3.1 Qualitative characterization of the bio-catalytic agent

Drop-collapse test and oil-spreading assay are qualitative and fast tests used for the prior evaluation of the surface activity of the bio-catalytic agent and its performance. Both of them tested a positive result indicating a satisfactory efficiency of the product. The evident cleaning and removal action of petroleum from the contaminated area (Petri dish) is intimately associated with the amphiphilic nature of the non-ionic surfactant present in this bio-catalyst, which reduces the surface tension and favored the miscibility between two different polarities substances such as water and oil.

### 3.2 Emulsification assay (E_24_)

**Figure 1:**
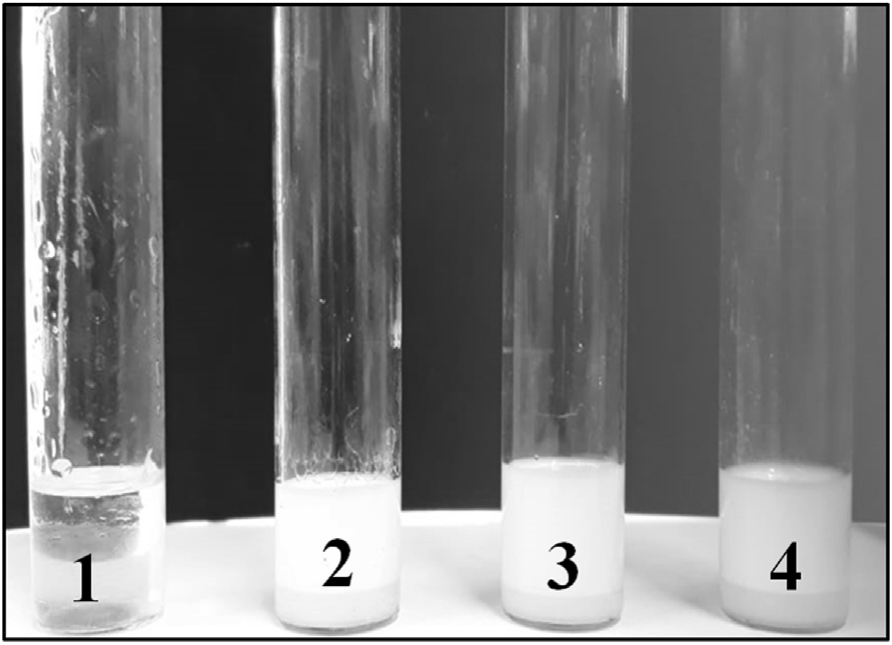
Test-tube used to measure the emulsification index (E_24_), (1). The first test-tube is the control sample whose composition is only 2 mL kerosene. (2), (3), (4). These test-tubes contain a mixture of 2 mL kerosene with 2 mL of bio-catalytic agent solution previously stirred in a vortex type agitator for 2 min.

Franzetti et al. (2010) reported that the emulsification process begins when there is enough accumulation of surfactant that forms a solution that contains tiny droplets of oils suspended in an aqueous medium. Figure 1 shows the set-up of the experiment carried out to evaluate the emulsifying activity of the bio-catalyst. After twenty-four (24) hours, the average emulsification index obtained from three independent measurements was 74.47% ± 5.55 as proof of the excellent tensoactive properties of the bio-catalyst studied. This value surpasses the acceptable emulsification index for a good surfactant of 40%, which buttresses the high product quality (Youseff et al., 2004); additionally, the high molecular weight of surfactants gives them the characteristic of efficient emulsifiers (Souza et al., 2014).

### 3.3 Critical Micelle Concentration

By means of this test was possible to determine the CMC of the bio-catalytic agent evaluated in this study. This concentration is known as the value from which begins the micelle formation (Souza et al., 2014). The minimal concentration of surfactant required to reduce the surface tension to its maximum extension, enhancing the oil solubility in the aqueous medium (Moro et al., 2018). As presented in Table 2, approximately 40 mg L^−1^ of bio-catalytic agent solution was necessary to reduce the surface tension of water from 73.1 to 29.0 mN m^−1^. These outcomes are comparable to the CMC values obtained for the most efficient surfactant tested by Moro et al. (2018). They also observed a high initial value of surface tension corresponds to water, followed by a significant decrease due to the presence of bio-catalyst in the solution. The CMC was then selected at the minimum value of surface tension obtained, henceforth the surface tension measurements were kept almost constant regardless of the increase in the concentration of bio-catalytic solution. These results render attractive this bio-catalytic agent, knowing that a surfactant is considered suitable when it is able to reduce the surface tension of water to 35 nN m^−1^ or less (Patowary et al., 2015). Zhang and Miller (1992) mention that the required concentration to diminish the surface tension of water from 71.2 mN m^−1^ to values below 40 mN m^−1^ varies between 1 and 200 mg L^−1^. The CMC of this surfactant is low, which means that a low concentration of the product can decrease the surface tension of water, favoring the biological availability of the hydrophobic substrate (petroleum hydrocarbons) to microorganisms and the interfacial surface reduction between the bacteria cell wall and hydrocarbon molecules.

**Table 2:** Surface Tension values measured at a given concentration of bio-catalytic agent.

Bio-catalytic Agent Concentration (mg L^−1^)	Surface Tension (mN m^−1^)
540	30.0 ± 0.08
430	30.2 ± 0.09
140	29.8 ± 0.16
70	29.4 ± 0.25
60	29.6 ± 0.11
50	29.8 ± 0.41
40	29.0 ± 0.10
30	29.2 ± 0.64
20	29.8 ± 0.70
10	29.2 ± 1.05
5	29.4 ± 1.23
0 (Distilled water without bio-catalyst nor oil)	73.1 ± 0.95

The concentrations tested in this study were above the CMC. This is based on some findings that conclude that the surfactant concentration must be above the respective CMC, to increase the solubilization/desorption of aliphatic or Polycyclic Aromatic Hydrocarbons (PAHs) from one medium to another (Zhu and Aitken, 2010), and achieve the maximum effect of the surfactant (Sajjadi et al., 2010).

### 3.4 Analysis of chemical and physical properties of seawater during the bioremediation

**Figure 2:**
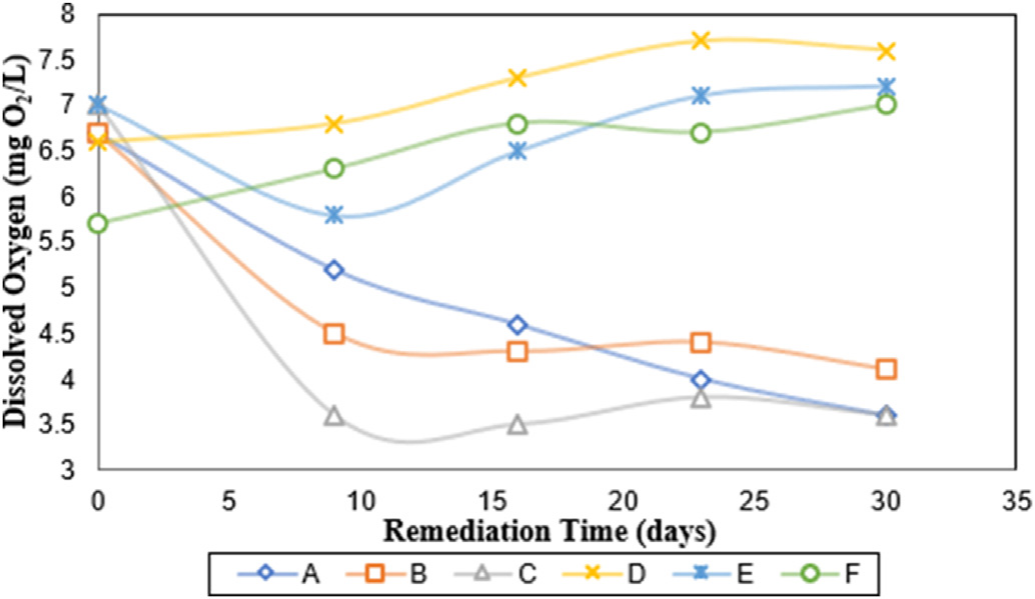
Variation of dissolved oxygen content with remediation time. (A) Seawater with oil (640 mg L^−1^); (B) Seawater with oil (640 mg L^−1^) and bio-catalytic agent solution (2167 mg L^−1^); (C) Seawater with oil (640 mg L^−1^) and bio-catalytic agent solution (10000 mg L^−1^); (D)Seawater with bio-catalytic agent solution (2167.39 mg L^−1^) and aeration system; (E) Seawater with oil (640 mg L^−1^), bio-catalytic agent solution (2167 mg L^−1^), and aeration system; (F) Seawater with oil (640 mg L^−1^), bio-catalytic agent solution (10000 mg L^−1^) and aeration system.

Figure 2 illustrates the variation of Dissolved Oxygen (DO) with respect to the remediation time for the polluted water sample in the six vessels. It was appreciated that the DO increased with remediation time for the samples labeled D, E, and F, where took place an effective degradation associated with the cracking of petroleum hydrocarbons. Hence, the indigenous microorganisms present in the medium do not need the same Oxygen concentration for their respiration process due to the reduction of organic matter.

The least DO value was observed in the control sample(A), which did not have the bio-catalyst solution or the aeration system. Likewise, the samples labeled B and C showed the same behavior as the sample labeled A. However, it is worth pointing out that after day sixteen (16) of the experiment, the DO content started rising in the samples labeled B and C. This event is related to bio-catalytic agent capacity to improve the Oxygen transfer and speed up the degradation of organic matter. The higher the Dissolved Oxygen level, the better the water quality and vice versa.

**Figure 3:**
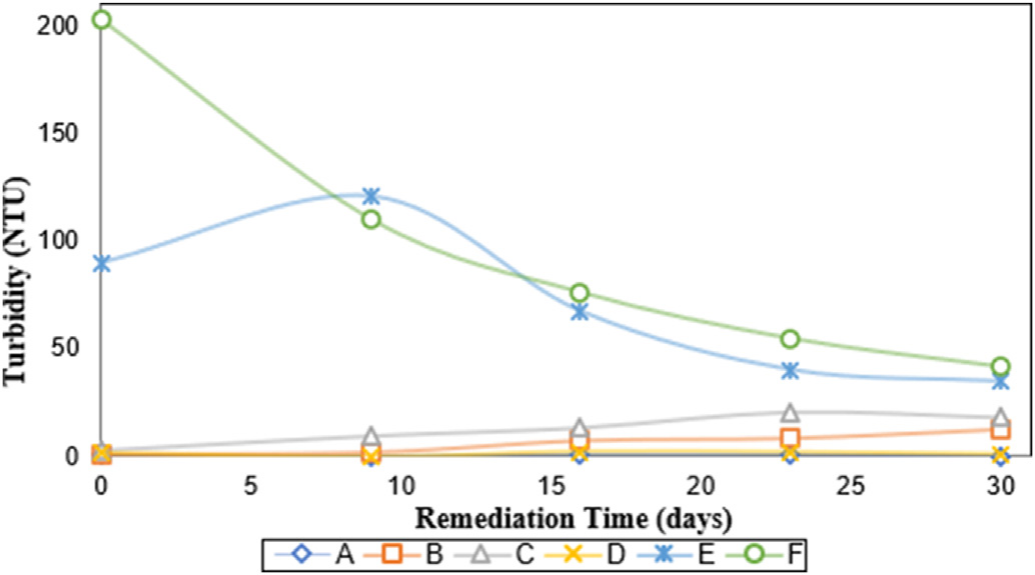
Variation of turbidity with remediation time. (A) Seawater with oil (640 mg L^−1^); (B) Seawater with oil (640 mg L^−1^) and bio-catalytic agent solution (2167 mg L^−1^); (C) Seawater with oil (640 mg L^−1^) and bio-catalytic agent solution (10000 mg L^−1^); (D) Seawater with bio-catalytic agent solution (2167.39 mg L-1) and aeration system; (E) Seawater with oil (640 mg L^−1^), bio-catalytic agent solution (2167 mg L^−1^), and aeration system; (F) Seawater with oil (640 mg L^−1^), bio-catalytic agent solution (10000 mg L^−1^) and aeration system.

Turbidity analysis displayed positive results for oil-polluted samples labeled E and F stimulated with the abio-catalytic agent solution and an aeration system. They achieved turbidity reduction values of 61.357% ± 0.053 and 79.623% ± 0.053, respectively (see Figure 3). Turbidity is inversely proportional to water quality. Therefore, it is valid to affirm that the sample labeled F displays better quality and performance than other studied samples during the remediation time.

**Figure 4:**
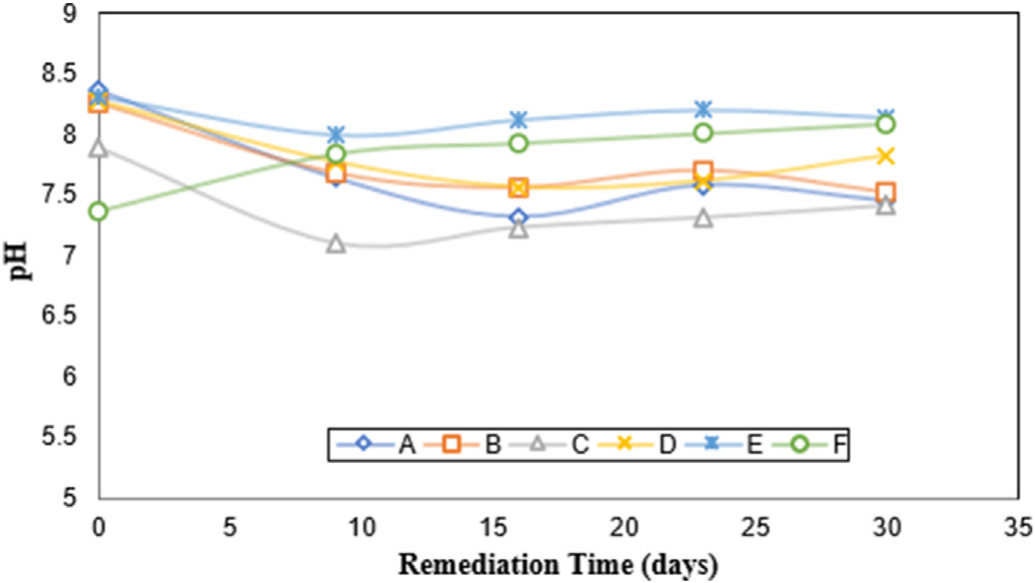
Variation of pH with remediation time. (A) Seawater with oil (640 mg L^−1^); (B) Seawater with oil (640 mg L^−1^) and bio-catalytic agent solution (2167 mg L^−1^); (C) Seawater with oil (640 mg L^−1^) and bio-catalytic agent solution (10000 mg L^−1^); (D) Seawater with bio-catalytic agent solution (2167.39 mg L-1) and aeration system; (E) Seawater with oil (640 mg L^−1^), bio-catalytic agent solution (2167 mg L^−1^), and aeration system; (F) Seawater with oil (640 mg L^−1^), bio-catalytic agent solution (10000 mg L^−1^) and aeration system.

From Figure 4, it was observed that samples labeled A, B, C, D, and E showed a similar tendency. In the beginning, was detected a decrease in the pH values linked to the possible decomposition of petroleum hydrocarbons to carbon dioxide. Nonetheless, from sixteen (16) days, these samples started increasing its pH value as proof of bioremediation (Anih et al., 2019). The pH of the sample labeled F exhibited a continuous rising in pH values, indicating that pollutant (petroleum hydrocarbons) in the water was decomposed to compounds that are more basic and less toxic (Amenaghawon et al., 2014; Obahiagbon and Aluyor, 2009).

**Figure 5:**
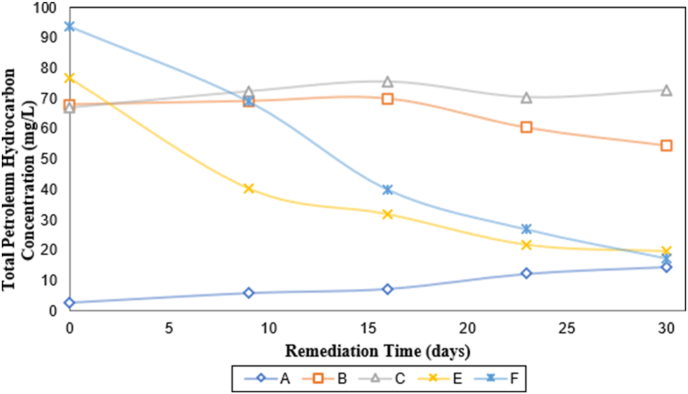
Variation of total petroleum hydrocarbon concentration with remediation time. (A) Seawater with oil (640 mg L^−1^); (B) Seawater with oil (640 mg L^−1^) and bio-catalytic agent solution (2167 mg L^−1^); (C) Seawater with oil (640 mg L^−1^) and bio-catalytic agent solution (10000 mg L^−1^); (E) Seawater with oil (640 mg L^−1^), bio-catalytic agent solution (2167 mg L^−1^), and aeration system; (F) Seawater with oil (640 mg L^−1^), bio-catalytic agent solution (10000 mg L^−1^) and aeration system.

The effect of remediation time on TPHC of the samples is presented in Figure 5. From this graphic is possible to evidence the TPHC decrease with remediation time for samples labeled E and F. This reduction is credited to the presence of a bio-catalyst solution and an aeration system which generates the formation of micro-bubble. These micro-bubbles are the result of aggregates of surfactant molecules with a loose molecular packing, which provokes a more favorable Oxygen mass transfer into an aqueous medium (Basařová and Zedníková, 2019). This phenomenon contributes to hasten the crude oil hydrocarbons biological degradation rates.

The effect of incorporation micro-bubbles was positive for the remediation of diesel-contaminated soil carried out by Ayele et al. (2020). They evaluated the influence of aeration system on diesel-pollutant removal efficiency and compared it with a non-aeration system. They noticed that the aeration doubtless help to accelerate the contaminants degradation rates. With aeration, they achieved to increase the diesel removal efficiency from 12% to 25% depending on the particle size of soil, organic matter level, and age of contamination. This wide gap of removal efficiency could be explained by taking into account that as airflow rate increases more hydrophobic micro-bubbles will be created with the large interfacial surface area, which will enhance the contact surface area between the pollutants and the surfactant solution helping it to separate pollutants from the contaminated site (Agarwal and Liu, 2017).

Furthermore, Parhizcar et al. (2015) in their investigation comment that non-ionic surfactants produce more stable and smaller bubbles than anionic or cationic surfactants. This is linked to the presence of a larger hydrophilic group and therefore, the wettability of the channel wall surface is affected differently. This triggers the more efficient absorption onto hydrophobic surfaces than onto hydrophilic ones (Rosen and Kunjappu, 2012).

The analysis of the variation of TPHC for the control sample (A), which had neither the bio-catalyst solution nor aeration system, allowed us to observe low TPH concentration values at the beginning of the experiment due to the absence of the bio-catalyst solution in the sample. This led to no reduction of surface tension between petroleum-seawater, affecting the miscibility between the phases and the emulsion formation. Afterward, the measured concentrations of TPH trend towards increasing.

On the other hand, samples labeled B and C, in the beginning, exhibited an increasing tendency of TPHC. However, until sixteen-day, a TPHC reduction pattern for sample B was identified, while for sample C was clear to detect an initial decreasing value followed by a slight increase of TPH concentration. If the experiment had continued, it would be expected to see a diminishing of TPHC in the sample labeled B and values below 72.2 mg L^−1^ (last registered value) for sample labeled C.

The increase in the concentration of petroleum hydrocarbons for samples labeled B, C and A is associated with the acclimatization or adaptation time required by the autochthonous microorganisms due to the nutrients (bio-catalyst) and contaminants (crude oil) added. It is presumably that these samples have required much more time than samples labeled E and F to assimilate the new environment, especially control sample (A) that did not have bio-catalyst. This, in turn, obstacle the degradation process. Also, another but less probable reason could have been the evaporation of water due to a failure in the hermetic sealing of the vessels.

It should be pointed out that bioremediation of petroleum hydrocarbons-polluted ecosystems is usually limited due to the narrow diversity of autochthonous microflora and the scarcity of specific indigenous microbes for each type of petroleum hydrocarbons (Ron and Rosenberg, 2014). This event is sharply related to the behavior of the samples above mentioned, regarding the influence of the petroleum hydrocarbons-degrading microorganisms on crude oil removal efficiency. According to Atlas, 1991a, Atlas, 1991b, the quantity of petroleum-degrading microorganisms in a non-polluted medium comprises less than 0.1% of the total population. Despite that, this percentage could ascend to 10% of the total population in petroleum-contaminated ecosystems, even if it represents the decreasing microbial diversity of the natural environment (Atlas, 1991b; Gorvanyov, 2015).

This information could be validated when the aerobic mesophilic bacteria were counted in the aerated samples labeled E and F. Before contaminating the samples, the quantification method displayed values below the quantification limit, but at thirty (30) day of the experiment, the result obtained was 12 CFU 100 mL^−1^.

As we expected, the stimulated sample labeled F reached the highest %RTPH with a rough value of 81.537 ± 0.015, followed by 74.446 ± 0.015 corresponding to sample labeled E. These values are in the average range of efficiencies reported by Anih et al. (2019), whose investigation aimed to study the effect of nutrients on bioremediation of polluted crude-oil water. The results showed that the least TPHC removal efficiency was 66.1% for the control sample which had neither nutrients nor microbes added to it, and the highest TPHC reduction was achieved by a sample that comprised NPK fertilizer as the bio-catalyst, external microbes and was subjected to an aeration system. Furthermore, it is quite important to mention that Dell’Anno et al. (2018), based on the study done by Cheng et al. (2004), suggest that the combined use of chemical surfactants and bio-catalytic agents produce a symbiotic effect which can improve the toxic hydrocarbons removal efficiencies, including those more complicated to degrade as PAHs. In our case study, this product contains a non-ionic surfactant from plants, which could explain the rapid and effective TPH removal from the studied matrices of seawater.

For samples labeled B and C, there were no positive values of petroleum hydrocarbon removal for samples labeled B and C, considering their composition and experimental conditions, except for days twenty-three (23) and thirty (30), when sample labeled B accomplished an average percentage removal of 19.706% ± 0.015.

The %RTPH could have been improved by applying the bio-augmentation method and even better bio-stimulation and bio-augmentation approaches simultaneously. The addition of nutrient and scarce co-substates to stimulate the existing microorganisms and bringing new individual strain of microorganism or consortium of microbial strains in the medium can increasingly boost the bioremediation results. Many researchers have proven that an array of a strain of microorganisms is more potential than individual cultures for metabolizing/degrading a complete group of hydrocarbons (Deppe et al., 2005; Deziel et al., 1996; Varjani et al, 2013, 2015).

In the case of petroleum which is a mixture of complex and straightforward hydrocarbons, its simpler compounds can be degraded by a wide variety of bacteria, but the ability to degrade complex compounds (such as PAHs, resins, and asphaltenes) is found in very few species (Varjani, 2017). That is why a bacterial sp. specializes in the utilization of few hydrocarbons as a preferred food source while the consortium gives a synergistic effect (Perussitti et al., 2003; Sugiura et al., 1996; Varjani et al., 2015, 2013). This technique has achieved TPH removal efficiency values where the difference grows from 95.54% to 99.09% (Anih et al., 2019).

**Figure 6:**
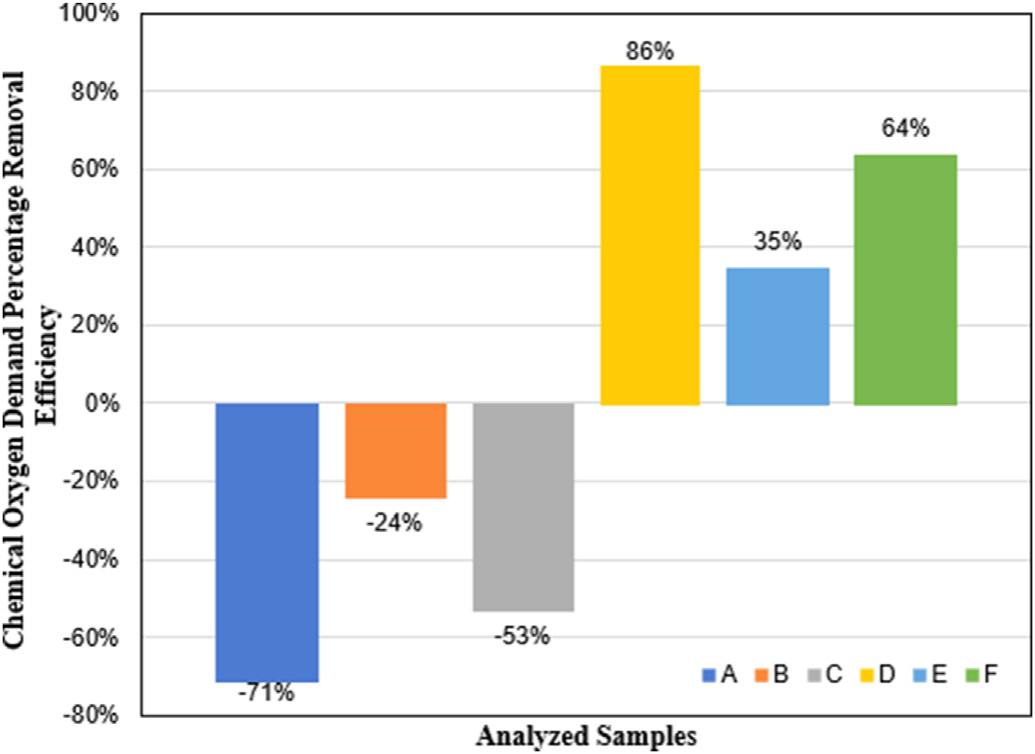
Percentage of Chemical Oxygen Demand removal (A) Seawater with oil (640 mg L^−1^); (B) Seawater with oil (640 mg L^−1^) and bio-catalytic agent solution (2167 mg L^−1^); (C) Seawater with oil (640 mg L^−1^) and bio-catalytic agent solution (10000 mg L^−1^); (D)Seawater with bio-catalytic agent solution (2167.39 mg L-1) and aeration system; (E) Seawater with oil (640 mg L^−1^), bio-catalytic agent solution (2167 mg L^−1^), and aeration system; (F) Seawater with oil (640 mg L^−1^), bio-catalytic agent solution (10000 mg L^−1^) and aeration system.

The COD removal efficiency of studied samples is depicted in Figure 6. All the seawater matrices showed a similar trend to the variation of TPH concentration with remediation time. The addition of bio-catalyst as a source of nutrients and the indigenous microbe consortium proved to be efficient enough to biodegrade aliphatic petroleum hydrocarbons, which are the most abundant compounds in the crude oil used in this study.

The highest %RCOD was 64.539 ± 0.125, and it was attained by sample labeled F, which had the most concentrated bio-catalyst solution, followed by sample labeled E which had a removal efficiency of 35.325% ± 0.125 for COD at the end of the remediation time. These results suggest the direct relationship between the concentration of this bio-catalyst solution and the fast capability to degrade the organic matter present in a contaminated environment.

The effect of the bio-catalyst on the bioremediation process was also assessed in the absence of the pollutants (crude oil hydrocarbons). From the beginning of the test, the sample labeled D presented a rapidly decreasing of COD values. This strengthens the premise that this product can amend bioremediation conditions, speed up bio-restoration rates, and improve Oxygen transfer to the water. These properties allowed this sample to end the experimentation with a %RCOD of 86.949 ± 0.125.

## 4 Conclusion

The effectiveness of a commercial bio-catalytic agent used on bioremediation of Total Petroleum Hydrocarbon-contaminated seawater was evaluated in this study. The analyzed bio-catalyst showed positive outcomes for the drop-collapse test and oil-spreading assay. The measurement of emulsification activity (E_24_) and Critical Micelle Concentration (CMC) displayed values of 74.47% and 40 mg L^−1^, respectively. All these values were higher than the satisfactory values reported in literature confirming the good quality of the product.

The quality values obtained were corroborated through the study of the degradation ability of the bio-catalyst. It enhanced the remediation process to different extents. The decrement of COD, turbidity, and DO content was noticeable in the crude oil-contaminated samples with bio-catalytic agent solution added and subjected to aeration systems. The highest TPH removal efficiency was reached by the sample labeled F, which contained 640 mg L^−1^ of petroleum and 10000 mg L^−1^ of bio-catalyst solution. The significant reduction of 81.537% allowed us to recognize this sample as the best water quality of the analyzed samples.

Furthermore, it was determined that agitation and aeration systems have an essential effect on the bioremediation process. As a matter of that, TPH removal efficiencies for aerated samples were in a range of 70%–82%. At the same time, those not subjected to an agitation system achieved only a near value of 20%. Unequivocally, catabolic cooperation between groups of microorganisms is important during the bioremediation process, because sometimes the complete petroleum hydrocarbons degradation by an only microorganism is not possible. This buttresses the preference of many investigators to apply the bio-augmentation method, or the bio-stimulation and bio-augmentation simultaneously with a consortium of microbial strains belong to different genera to attain best water quality and optimal results during remediation of crude oil-contaminated sites.

## Declarations

### Author contribution statement

Glenda Teran-Cuadrado: Conceived and designed the experiments; Performed the experiments; Analyzed and interpreted the data.

Efrain Polo-Cuadrado: Analyzed and interpreted the data; Contributed reagents, materials, analysis tools or data; Wrote the paper.

### Funding statement

This research did not receive any specific grant from funding agencies in the public, commercial, or not-for-profit sectors.

### Data availability statement

No data was used for the research described in the article.

### Declaration of interests statement

The authors declare no conflict of interest.

### Additional information

No additional information is available for this paper.
